# Bacterial *lux*-Biosensors for Detecting Specific Cell Responses to Membrane Damage

**DOI:** 10.3390/bios15120780

**Published:** 2025-11-26

**Authors:** Vladimir A. Plyuta, Evgeny Y. Gnuchikh, Anastasiia A. Gorbunova, Veronika D. Udovichenko, Kristina A. Sinyakova, Daria E. Sidorova, Olga A. Koksharova, Sergey V. Bazhenov, Olga E. Melkina

**Affiliations:** 1 Complex of NBICS Technologies, National Research Center “Kurchatov Institute”, Kurchatov Sq. 2, 123182 Moscow, Russia; gnuchih_evgeniy@mail.ru (E.Y.G.); misenok1@gmail.com (D.E.S.); koksharova@belozersky.msu.ru (O.A.K.); compleanno@mail.ru (O.E.M.); 2 Kurchatov Center for Genome Research, National Research Center “Kurchatov Institute”, Kurchatov Sq. 2, 123182 Moscow, Russia; 3 A.P. Nelyubin Institute of Pharmacy, I.M. Sechenov First Moscow State Medical University of the Ministry of Health of the Russian Federation (Sechenov University), Trubetskaya Str. 8-2, 119991 Moscow, Russia; anastasiia.gorbunova727@gmail.com; 4 Faculty of Chemical Technology and Biotechnology, Moscow Polytechnic University, Bolshaya Semyonovskaya Str. 38, 107023 Moscow, Russia; v.udov04@mail.ru (V.D.U.); sin.cristina1@yandex.ru (K.A.S.); 5 A.N. Belozersky Institute of Physico-Chemical Biology, Lomonosov Moscow State University, Leninskie Gory 1-40, 119992 Moscow, Russia; 6 Moscow Center for Advanced Studies, Kulakova Str. 20, 123592 Moscow, Russia; bazhenov1994@gmail.com

**Keywords:** whole-cell biosensor, *pspA*, *E. coli*, *B. subtilis*, luminescence, membrane damage, membrane permeabilization

## Abstract

Whole-cell biosensors represent one of the tools used for assessing the effects of various agents on living cells. Here we have constructed and tested whole-cell *lux*-biosensors to detect membrane damage in both Gram-negative and Gram-positive bacteria using the stress-inducible promoter of the *pspA* gene from *Escherichia coli* and *Bacillus subtilis* fused to the *lux* genes from *Photorhabdus luminescens*. These biosensors increase their luminescence in response to treatment with a number of known membrane-damaging compounds, such as ethanol, Triton X-100, polymyxin B, dimethylsulfoxide (DMSO) and melittin. *E. coli-* and *B. subtilis*-based biosensors demonstrated differences in response to the action of the same membrane-damaging agent. Thus, ethanol and polymyxin B specifically induced the *pspA* promoter in both *lux*-biosensors, but the induction amplitude was higher in the *E. coli*. Triton X-100 and melittin specifically induced the *pspA* promoter exclusively in *B. subtilis* cells, while DMSO induced it only in *E. coli* cells. This indicates a difference in the stress response of the Psp system to membrane-damaging agents in *E. coli* and *B. subtilis* cells. Thus, we demonstrated the functionality and efficiency of the constructed *lux*-biosensors and, using them, showed that some of the tested compounds are able to specifically activate Psp stress response systems in case of membrane damage.

## 1. Introduction

Throughout their life cycle, bacteria can undergo cell membrane destruction under the influence of variety of factors, including physical effects such as shear stress and cavitation, chemicals such as antibiotics (polymyxins, daptomycin), antimicrobial peptides, and host defense mechanisms such as lysozyme. Reactive oxygen species (ROS) can damage membranes as result of lipid peroxidation, while enzymes such as phospholipases can alter membrane fluidity. Physical stress (such as exposure to ultrasound) can also lead to leakage of internal components of the cell, such as ATP and DNA, indicating membrane damage [1,2,3,4].

Whole-cell biosensors are one of the tools capable of assessing cell damage by identifying specific responses of living cells to stress. They are used in the field of genetic engineering and biotechnology as a convenient tool designed not only to detect biologically active substances (especially antibiotics) and toxic agents in environmental monitoring or food safety tests, but also to determine the mechanisms of action of new compounds and nanoparticles in living cells [5,6,7,8,9]. Potential applications of biosensors in agriculture are also mentioned: for example, a whole-cell biosensor immobilized in hydrogel matrices has successfully identified pathological processes in agricultural crops (potatoes and citrus fruits) infected with pathogens; a biosensor system consisting of an alginate-based hydrogel embedded with bacteria and a luminescence-detecting sensor demonstrated significant potential for early detection of crop infections due to the detection of volatile organic compounds [10]; *E. coli*-based *lux*-biosensors were used to assess the genotoxicity of herbicides and pesticides, as well as their ability to induce stress responses (oxidative stress; damage to proteins, membranes, and other components) [11].

*Lux*-biosensors are bacterial cells that contain a transcriptional fusion of a regulatory system (promoter-operator region) and a cassette of reporter genes, e.g., from *Photorhabdus luminescens*. We chose the phage shock protein (Psp) system, which is found in both Gram-negative and Gram-positive bacteria, as a regulatory system [12,13]. Although the Psp system is conserved across many bacterial species, its specific components and regulation can be modular, adapting to the unique cell envelope (wall) structures and lifestyles of various bacterial lineages.

It is known that envelope homeostasis is vital for cell functioning. In turn, envelope stress responses initiate a preventative and/or corrective response, e.g., the bacterial Psp response, which protects the bacterial membrane under various extracytoplasmic stress conditions. Although many different stimuli induce the Psp response (e.g., heat and osmotic shock, ethanol treatment, blocking of Sec machinery for protein export, improper localization of secretin, inhibition of lipid biosynthesis, addition of uncouplers and proton ionophores), a common cause is perturbation/disruption of the inner membrane integrity and, consequently, dissipation of the proton motive force (PMF) [12,13,14,15].

The Psp system of Gram-negative bacteria consists of a ‘minimal’ module of four genes: *pspF* and *pspABC* [12,13]. The expression of the *pspABC* operon is controlled by the regulatory protein PspF, which is encoded upstream of *pspA* [14]. The central component of the Psp system is the peripheral plasma membrane binding protein, PspA, belonging to the IM30 protein family. In the normal state, the PspA–F complex inhibits the *pspA* promoter; under stress, the complex dissociates, and the released PspF activates the *pspA* promoter. In bacteria, the Psp response and PspA-like proteins are involved in protein translocation, virulence, and antimicrobial resistance, which affect the cell wall or reorganize the membrane architecture [13,15].

The *B. subtilis* genome encodes two PspA paralogs: PspA and LiaH. The gene annotated as *pspA* is regulated by the accessory sigma factor σ^W^, which directs transcription to genes involved in protecting the cell membrane from the permeabilization by lantibiotics (antimicrobial peptides that are produced by Gram-positive bacteria) [16]. The σ^W^ regulon includes *sppA*, which encodes a signal-peptide peptidase that presumably destroys antibiotics, and the operons *yceCDEFGHI* and *yvlABCD* [17]. The gene encoding LiaH protein is located in the *liaIHGFSR* operon, which responds to cell wall stress and forms oligomeric ring structures that maintain membrane integrity when the cell is exposed to stressors such as lantibiotics. Unlike the *pspA* gene, which is regulated by sigma factor σ^W^, *liaH* is controlled by the two-component system LiaRS, which activates the *liaIH* operon in response to cell wall stress [18,19,20]. The two PspA paralogs of *B. subtilis* show functional similarities; moreover, *liaH* and *pspA* partially complement each other [17].

In this study, we present whole-cell *lux*-biosensors based on *E. coli* and *B. subtilis* cells, constructed using a stress-inducible promoter of the *pspA* gene, to detect bacterial response to membrane perturbations (e.g., loss of proton motive force, lipid reorganization).

## 2. Materials and Methods

### 2.1. Strains and Plasmids

Bacterial strains and plasmids used in the current work are presented in Table 1. *E. coli* MC1061 cells were used for constructing biosensor plasmids. *E. coli* MG1655 cells were used for obtaining *E. coli*-based biosensors.

### 2.2. Enzymes and DNA Manipulation

Plasmid DNA was isolated by the QIAprep Spin Miniprep Kit (Qiagen, Hilden, Germany). The *E. coli* cell transformation with hybrid plasmids, agarose gel electrophoresis, and isolation of plasmid and total DNA was performed according to [23]. A restriction digest was carried out using the NcoI, SacI/SpeI, BamHI, ApaI, XhoI, KpnI restriction enzymes (Thermo Fisher Scientific, Waltham, MA, USA). Ligation was conducted with the use of Gibson Assembly, prepared according to [24], or T4 DNA ligase (Thermo Fisher Scientific, Waltham, MA, USA). *B. subtilis* cells were transformed according to [25].

### 2.3. Chemicals

Enzymes for Gibson Assembly preparation were purchased from NEB (Ipswich, MA, USA). Growth media were from Helicon (Moscow, Russia). Oligonucleotides were made by Syntol (Moscow, Russia). All chemicals were of analytical purity. Antibiotics (chloramphenicol and ampicillin) were obtained from Biopharm (Moscow, Russia); Trimethoprim (T7883; purity ≥ 98.5%)—Sigma-Aldrich Chimie GmbH (Steinheim, Germany).

Triton X-100 (≥98% purity) was purchased from Serva Electrophoresis GmbH (Heidelberg, Germany). Ethanol (96%) was purchased from LLC Donskoy (Tula region, Kimovsky district, Epifan, Russia), Dimethyl sulfoxide (DMSO; 99.9% purity)—Sigma-Aldrich Chimie GmbH (Steinheim, Germany), and o-nitrophenyl-3-D-galactoside (ONPG; purity ≥ 99%)—Chem-Impex International, Inc. (Wood Dale, IL, USA).

Antimicrobial peptides Melittin (>98% purity) and Polymyxin B (AppliChem GmbH, Darmstadt, Germany) were provided by Dr. Pavel V. Panteleev (M.M. Shemyakin and Yu. A. Ovchinnikov Institute of Bioorganic Chemistry, Russian Academy of Sciences, Moscow, Russia).

All test solutions and their dilutions were prepared immediately before use. The required concentrations of the stock solutions of ethanol, Triton X-100, Melittin, and Polymyxin B were obtained by dissolving the above compounds in distilled water; ONPG—in phosphate-buffer saline.

### 2.4. Constructing of Biosensor Plasmids

(a) Construction of pPspA plasmid for *E. coli*-based biosensor strain

pDEW201 was linearized by the BamHI/KpnI restriction enzymes. P*pspA* promoter region was amplified by PCR with primers PspAF/PspAR (see Table A1 in the Appendix A) and *E. coli* MG1655 genomic DNA as a template. Ligation of BamHI/KpnI-treated promoter-containing DNA fragment with linearized vector was conducted with the use of T4 DNA ligase. Resulting plasmid pPpspA::lux was used for transformation of *E. coli* MG1655 cells for obtaining *E. coli*-based biosensor strain. The design of *lux*-biosensor is developed according to [26].

(b) Construction of pMW-PpspA plasmid for *B. subtilis*-based biosensor strain

**Figure A1:**
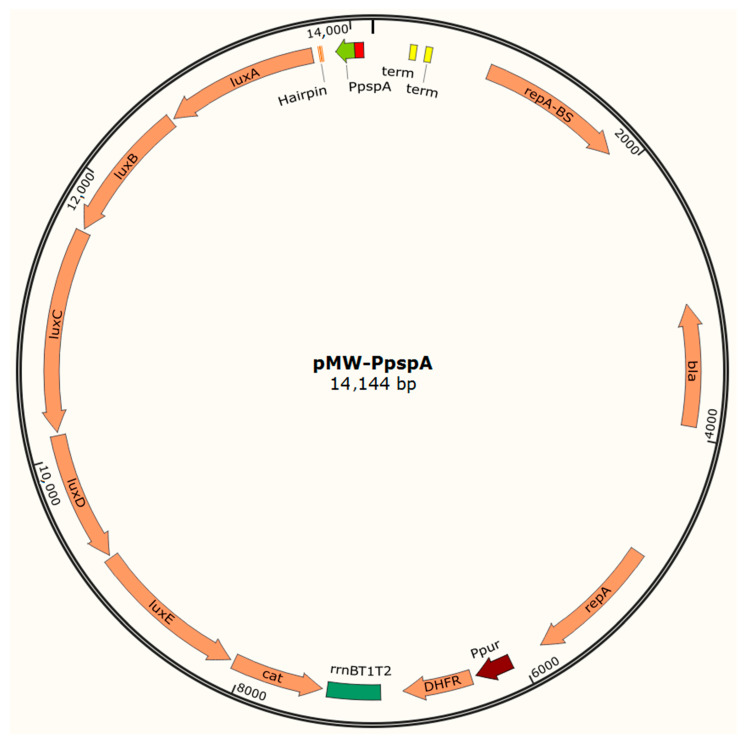
Structure of the pMW-PpspA plasmid. Created with SnapGene.

During the preparation of pMW-PpspA plasmid a series of intermediate plasmids was constructed. The list and stages of the construction of intermediate plasmids based on the methods specified in the works of Spizizen et al. (1958) and Gnuchikh et al. (2021) are described in Appendix A [25,27]. Briefly, the constructed plasmid pPL_ABCDExen-cat-hairpin was linearized using XhoI/KpnI restriction enzymes. DNA fragment with P*pspA* promoter region was amplified by PCR using the P7/P8 primers (Table A1) and *B. subtilis* 168 genomic DNA as a template and treated with XhoI/KpnI. The ligation was performed using T4 DNA ligase. The resulting plasmid was named pMW-PpspA (Figure A1 in Appendix A). *E. coli* MC1061 cells were used for the primary transformation and isolation of plasmid DNA. The pMW-PpspA plasmid isolated from *E. coli* cells was used to transform *B. subtilis* 168 cells in order to obtain a biosensor strain.

### 2.5. Culture Medium and Growth Conditions

*E. coli* cell cultures were grown at 30 °C in Lysogeny Broth (LB). *B. subtilis* cells were grown at 30 °C in tryptone broth as described by Gnuchikh et al. [28]. The LB medium was composed of 1% tryptone, 0.5% yeast extract, and 1% NaCl; the tryptone broth —1.5% tryptone and 0.5% NaCl. To obtain solid medium, agar was added to a final concentration of 1.5%. The LB media were supplemented with 100 µg/mL ampicillin or 10 µg/mL chloramphenicol; the tryptone broth media—with trimethoprim (10 µg/mL).

For experiments to determine the sensitivity of biosensors, overnight cultures were used to inoculate liquid LB or tryptone broth to an optical density (OD) of 0.01; the resulting cultures were grown with continuous agitation at 37 °C to a final OD of approximately 0.2 for *E. coli* and 0.4 for *B. subtilis*. The OD of cell suspensions was measured using the KFK-3 photometer (ZOMP, Moscow, Russia).

### 2.6. Measurement of In Vivo Luminescence

Cell culture was transferred to a 96-well plate at 200 µL of culture/well (black-walled, transparent flat bottom; cat. #665096 Greiner Bio-One, Frickenhausen, Germany). The tested compounds at various concentrations were added to induce the *pspA* promoter, and immediately afterward, the plate was placed into the reader. The first measurement corresponds to time “0”, which actually occurs approximately 3–10 min after the addition of the various chemicals. For each sample, 5 wells were used as technical replicates. A well with sterile media was used as a blank. The plates were incubated at 30 °C with double-orbital shaking at 400 rpm using a CLARIOstar Plus luminometer (BMG Labtech, Ortenberg, Germany). OD_600_ and luminescence were measured every 15 min. The luminescence emission filter was not used. The gain of the photomultiplier was automatically controlled by the enhanced dynamic range function. The measured values were normalized to an accumulation time of 1 s.

The data obtained were analyzed using the MARS software (version 3.42 R5). The blank values were subtracted from the raw values of OD_600_ and luminescence values expressed in relative luminescence units (RLU). The adjusted RLU reads at each time point were divided by the corresponding OD_600_ values to normalize RLU to the number of cells in each well. The average RLU/OD_600_ values and standard deviations were calculated and plotted depending on time.

### 2.7. Determination of the lux-Biosensors Characteristics

The main characteristics of the obtained biosensors, induction factor (IF), response time, threshold concentration, and working concentrations range were evaluated.

Induction factor (IF) was determined by the formula IF = I_t_/I_k_, where I_k_ is the average value of the bioluminescence intensity of the control sample (spontaneous luminescence of the bacterial culture without an inducer) at time *t*, and I_t_ is the average value of the intensity of bioluminescence of the test sample (luminescence of the bacterial culture in the presence of an inducer) at time *t*. The induction factor determines the strength of the biosensor response.

The induction start time (IST) is the time interval between the moment when the inducer is added (*t* = 0) and the moment *t* when the device detects a substantial increase in the bioluminescence signal in the experimental sample compared to the control sample (IF value is 2 or more). Significance of the difference between I_t_ and I_k_ was determined using one-way ANOVA analysis, followed by Tukey’s HSD (Honestly Significant Difference) post hoc test. The differences were considered statistically significant at *p* ≤ 0.05.

The threshold concentration was defined as the minimum concentration of the inducer at which IF reaches a value of 2.

Calculated IF values were the mean values ± standard deviations (SD) for *n* = 3 (three biological replicates were conducted, in each of which at least three parallel wells (technical replicates) were performed for each sample).

### 2.8. Permeabilization of Inner Membranes of E. coli ML-35p

The bacterial strain *E. coli* ML-35p is constitutive of cytoplasmic β-galactosidase, and lacks lac permease. Since this strain of *E. coli* lacks lactose permease, o-nitrophenyl-β-D-galactopyranoside (ONPG) cannot penetrate its inner membrane and be cleaved by cytoplasmic β-galactosidase to o-nitrophenol, unless permeabilization of the inner membrane occurs. ONPG cleavage produces a color change that can be measured spectrophotometrically at wavelengths of 405–420 nm.

The effect of known membrane disrupting compounds on the integrity of the inner membrane was determined using the *E. coli* ML-35p strain as described by Panteleev et al., 2018, with some modifications [29]. Briefly, bacteria were grown in TSB medium from a single colony, overnight at 37 °C. After three washings in phosphate-buffer saline (PBS), pH 7.4 (10 mM phosphate buffer, 0.1 M NaCl), the culture was diluted to 2.5 × 10^7^ CFU/mL in incubation buffer (PBS, pH 7.4 with 2.5 mM ONPG). The cell-free lysates were obtained as described by Kolev et al. 2021 with some modification [30]. The cells were disrupted via sonication on ice (pulse-on 30 s, pulse-off 30 s, 5 times), and the sonicated solution was centrifuged at 16,000× *g* force for 10 min at 4 °C to eliminate cell debris and unbroken cells. The clear supernatant extract was used to further evaluate the effect of the studied compounds on the enzymatic activity of β-galactosidase. The prepared cell culture (or cell-free lysate) was transferred to a 96-well plate at 150 µL of culture/well (black-walled, transparent flat bottom; cat. #665096 Greiner Bio-One, Frickenhausen, Germany). The tested compounds were added to the sample at various concentrations (the control sample was a biosensor culture/cell-free supernatant without the studied compounds), and immediately afterward, the plate was placed into the reader. The first measurement corresponds to time “0”, which actually occurs approximately 3–10 min after the addition of the various chemicals. A well with a sterile incubation buffer was used as a blank. The plates were incubated at 30 °C with double-orbital shaking at 400 rpm using a CLARIOstar Plus luminometer (BMG Labtech, Ortenberg, Germany). The absorbance was measured every 10 min at a wavelength of 420 nm. The data obtained were analyzed using the MARS software. The blank values were subtracted from the raw values of OD_420_.

For the *E. coli* ML-35p biosensor, the induction factor was determined by the formula IF = At/Ak. Ak is the average value of the absorption of the control sample measured at a wavelength of 420 nm (as an indicator of the permeability of the inner cell membrane of a bacterial culture without an inducer in the PBS buffer) at time *t*, and At is the average value of the absorbance at 420 nm of the test sample (as an indicator of the permeability of the inner cell membrane of the bacterial culture in the presence of an inducer in the PBS buffer) at time *t*. The statistically significant excess of At over Ak was assessed using a one-way ANOVA analysis. The differences were considered statistically significant at *p* ≤ 0.05. At least three biological replicates were performed (with at least five parallel wells for each sample (technical replicates)), and in all of them the curve pattern was similar.

## 3. Results

The whole-cell biosensors were created to detect membrane damage in bacteria by the fusion of stress-inducible promoter of the *pspA* gene of *E. coli* [14] and *B. subtilis* [31] with the *lux* genes of *P. luminescens* as a reporter. The effectiveness of the constructed *lux*-biosensors (response to membrane damage) was evaluated using known membrane-disrupting agents—ethanol, Triton X100, DMSO, as well as antimicrobial peptides polymyxin B and melittin in various concentrations (Table 2, Figure S1). The effects of toxicants on biosensor cells can have a dual effect, depending on their concentration. Moderate concentrations, which the cell can tolerate, trigger the activation of defense mechanisms, resulting in dose-dependent biosensor response. Exceeding a critical concentration results in cell death, metabolic inhibition, and/or luciferase denaturation, along with a decrease in luminescence. The concentrations of chemicals given in Table 2 caused dose-dependent response of biosensors. Chemicals can induce rapid changes in luminescence, causing either its inhibition or induction, with effects being especially pronounced when acute toxicity occurs. Therefore, differences in luminescence values will be noticeable already at time “0” compared to the control sample, due to a technical delay of several minutes between the addition of chemicals to cells and the start of measurements. To confirm that the activation of the biosensors (increase in their luminescence) is caused by the activation of the *pspA* promoter and is not due to a general effect on the cell or its other individual elements, control experiments were conducted. The same chemicals were added to the *E. coli* MG1655 pDlac [32] and *B. subtilis* 168 pPfbaA_ABCDExen cells carrying plasmids with transcriptional fusion of the *lux* genes with constitutive promoters (Figure S2). Exposure to concentrations higher than indicated in Table 2 led to a significant inhibition of biosensor growth and/or the luminescence reaction itself, which did not allow us to reliably determine the effect of these concentrations on the induction of the stress-responsive *pspA* promoter. Graphs of the luminescence of *lux*-biosensors in the presence of different concentrations of inducers depending on the incubation time are shown in Figures 2–5. (Two concentrations are given in each graph: the threshold concentration and the concentration at which the highest IF was achieved or the highest concentration in the tested range).

The specificity of the constructed biosensors was tested using agents that cause oxidative damage, such as hydrogen peroxide and paraquat [26,33], as well as the antibiotic anhydrotetracycline (binds to the bacterial ribosome, inhibiting protein synthesis and preventing the translation process) [34] as negative controls. The addition of these chemicals did not increase the luminescence of the P*pspA*-based *lux*-biosensors (Figure S3), which means that their action did not lead to specific induction of the stress-sensitive *pspA* gene promoter.

### 3.1. The Effect of Ethanol on the lux-Biosensors

Ethanol is a well-known substance that causes membrane damage (destruction and liquefaction of the cell wall and membranes) and a decrease in the membrane proton potential [35].

The effect of ethanol on the constructed *lux*-biosensors *E. coli* MG1655 (pPpspA::lux) and *B. subtilis* 168 (pMW-PpspA) was evaluated in the concentration range of 0.4–8% and 0.5–5%, respectively. The maximum luminescence induction factor (IF) in these concentration ranges was ~34 and 5 for *E. coli* MG1655 (pPpspA::lux) and *B. subtilis* 168 (pMW-PpspA) strains, respectively (Table 2).

**Figure 1:**
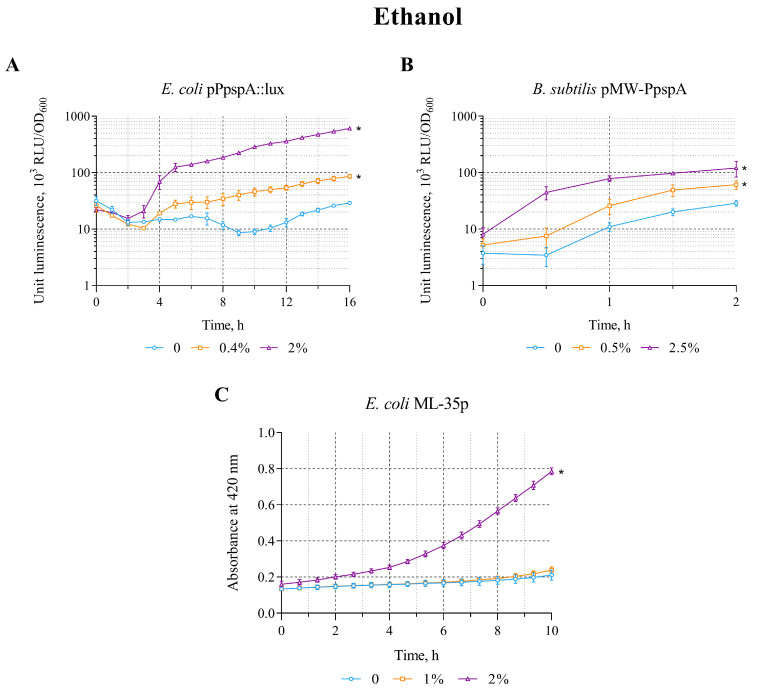
The response of biosensor strains to ethanol. (**A**) *E. coli* MG1655 (pPpspA::lux), (**B**) *B. subtilis* 168 (pMW-PpspA), and (**C**) *E. coli* ML-35p. (**A**,**B**)—The curves show the change in luminescence units of the biosensor cell response over time, which is expressed as the ratio of the average values of light emission (in Relative Light Units, RLU) measured at time t to its optical density (OD_600_) in the presence of various concentrations of ethanol. (**C**)—The kinetics of changes in the permeability of the cytoplasmic membrane of *E. coli* ML-35p during incubation with different concentrations of ethanol (1 and 2%) was determined by measuring the formation of o-nitrophenol at 420 nm after hydrolysis of the chromogenic marker ONPG by cytoplasmic bacterial β-galactosidase. The control measurements were carried out without the use of ethanol. All values were mean ± standard deviations (SD) for *n* = 3 (biological replicates). Significant differences were determined using one-way ANOVA followed by Tukey’s HSD post hoc test (*p* ≤ 0.05). Asterisks (*) indicate significant differences compared to the control sample (without chemicals). The same designations are used in the following figures.

A graphical representation of the effect of ethanol on biosensors is shown in Figure 1. Ethanol (0.4 and 2%) increased the bioluminescence intensity of *E. coli* strain MG1655 (pPpspA::lux) (Figure 1A). Luminescence induction started gradually after ~2.5 h of incubation (induction start time; IST) and after 6 h IF reached a value of 8.2 for a sample with a 2% ethanol content. Treatment of *B. subtilis* 168 (pMW-PpspA) cells with ethanol (0.5 and 2.5%) also led to a gradual increase in bioluminescence (Figure 1B). It should be noted that for the *B. subtilis*-based biosensor, the induction start time was shorter (IST = 30 min) compared with the stress response caused by membrane damage in the *E. coli*-based *lux*-biosensor. In both cases, the effect of ethanol in the studied concentration ranges led to activation of the P*pspA* promoter (it triggers a cascade of protein interactions that stabilizes the bacterial cell membrane).

Furthermore, the effect of ethanol on the integrity of the bacterial cytoplasmic membrane was tested using the *E. coli* strain ML-35p. Ethanol has been shown to damage the inner membrane, starting at a concentration of 2% (Figure 1C). The response amplitude gradually increased to a value of 3 with an induction start time of ~30 min.

The data obtained clearly demonstrate that ethanol not only causes permeability/disruption of the cytoplasmic membranes of *E. coli* ML-35p, but also induces specific bacterial defense systems in response to the stress it causes (which is consistent with the literary data [35,36]).

### 3.2. The Effect of Triton X-100 on the lux-Biosensors

The detergent Triton X-100 is widely used in laboratories to increase the permeability of living cell membranes [37,38].

The effect of triton X-100 on the *lux*-biosensors was tested in the concentration range of 40–400 µg/mL (*E. coli* MG1655 (pPpspA::lux)) and 20–200 µg/mL (*B. subtilis* 168 (pMW-PpspA)), respectively. Triton X-100 in the studied concentration ranges increases the bioluminescence intensity of only the *B. subtilis* 168 (pMW-PpspA) strain; the maximum luminescence induction factor (IF) was ~7 (Table 2).

**Figure 2:**
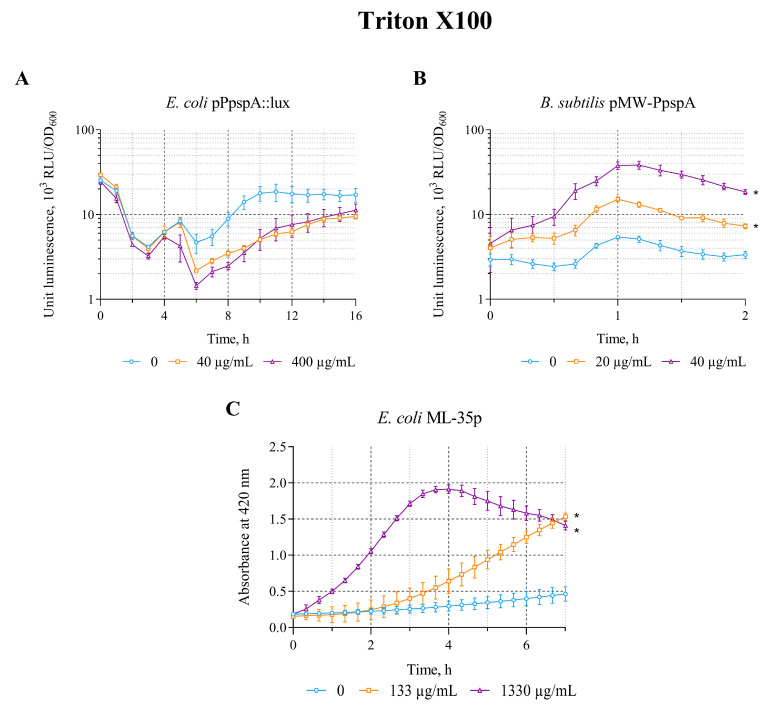
The response of biosensor strains to Triton X-100. (**A**) *E. coli* MG1655 (pPpspA::lux), (**B**) *B. subtilis* 168 (pMW-PpspA), and (**C**) *E. coli* ML-35p. All values were mean ± standard deviations (SD) for n = 3 (biological replicates). Significant differences were determined using one-way ANOVA followed by Tukey’s HSD post hoc test (*p* ≤ 0.05). Asterisks (*) indicate significant differences compared to the control sample (without chem-icals).

A graphical representation of the induction curves of the P*pspA* promoter of the biosensor strains over time with different concentrations of Triton X-100 is shown in Figure 2. Triton X-100 (20 and 40 µg/mL) increased the bioluminescence intensity of *B. subtilis* 168 (pMW-PpspA). The amplitude of the reaction gradually increased up to an IF value of 8 and reached a maximum after ~1 h of incubation, while the induction start time was ~30 min (Figure 2B). Concentrations above 200 μg/mL led to lysis of *B. subtilis* cells. Triton X-100 (40 and 400 μg/mL) did not induce the luminescence intensity of *E. coli* MG1655 (pPpspA::lux) strain and even caused a ~2–3-fold decrease after 4 h of incubation (Figure 2A). Although Triton X-100 did not induce the activity of the P*pspA* promoter in *E. coli* MG1655 (pPpspA::lux), it was able to induce the permeability of *E. coli* ML-35p cytoplasmic membranes in the concentration range of 133–4000 μg/mL (Table 2) with a maximum induction factor (IF) of 7. Thus, at 1330 μg/mL of Triton X-100, the response amplitude of the biosensor gradually increases to 7 times, and the induction start time is ~45 min (Figure 2C).

### 3.3. The Effect of DMSO on the lux-Biosensors

It is well known that dimethyl sulfoxide (DMSO; C_2_H_6_OS) exhibits various biological activities, including its effect on cell membrane permeability (e.g., membrane loosening, pore formation, and bilayer destruction) in living cells [39]. Therefore, in this work, an assessment of its effect on biosensors was carried out.

The opposite patterns (compared with the effect of Triton X-100) were observed when DMSO was applied to biosensors. DMSO in the concentration range of 0.5–5% induces the bioluminescence intensity of *E. coli* MG1655 (pPpspA::lux) strain. The average value of the maximum luminescence induction factor (IF) in these concentration ranges is ~10. DMSO does not induce luminescence of *B. subtilis* 168 (pMW-PpspA) and the permeabilization of *E. coli* ML-35p cytoplasmic membranes in the concentration ranges of 0.25–2.5% and 0.67–10%, respectively (Table 2).

**Figure 3:**
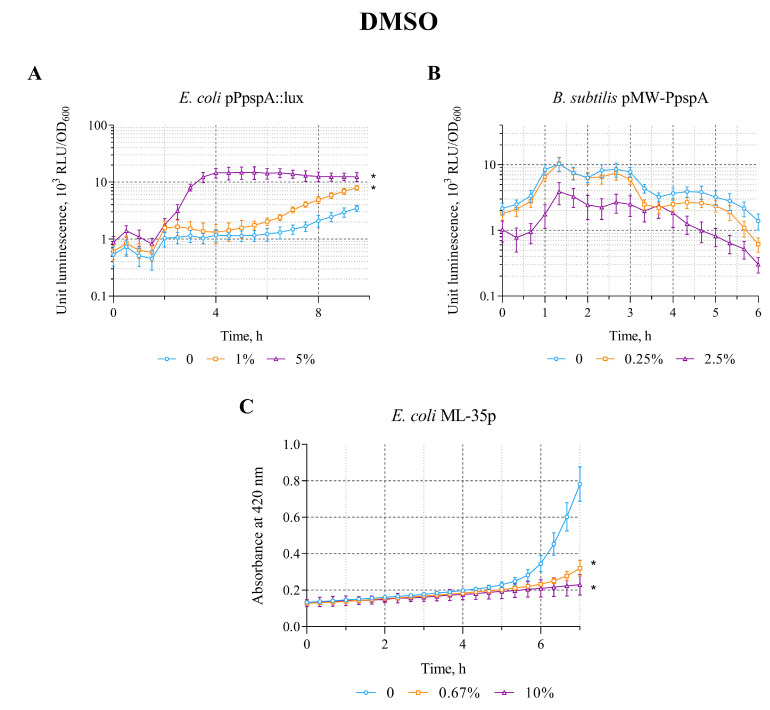
The response of the biosensor strains to DMSO. (**A**) *E. coli* MG1655 (pPpspA::lux), (**B**) *B. subtilis* 168 (pMW-PpspA), and (**C**) *E. coli* ML-35p. All values were mean ± standard deviations (SD) for n = 3 (biological replicates). Significant differences were determined using one-way ANOVA followed by Tukey’s HSD post hoc test (*p* ≤ 0.05). Asterisks (*) indicate significant differences compared to the control sample (without chem-icals).

A graphical representation of the effect of different DMSO concentrations on the induction of luminescence intensity of *lux*-biosensors depending on their incubation time is shown in Figure 3. DMSO (by 1 and 5%) increases the bioluminescence intensity of *E. coli* MG1655 (pPpspA::lux)—the response amplitude gradually increases up to an IF value of 13 and reaches a plateau after ~4 h of incubation with induction start time of ~110 min (Figure 3A). DMSO (0.25 and 2.5%) does not induce the luminescence intensity of *B. subtilis* 168 (pMW-PpspA) or even reduces it (Figure 3B). It should be noted that DMSO (0.67 and 10%) does not permeabilize the cytoplasmic membranes of the *E. coli* ML-35p strain (Figure 3C). Its action even leads to a decrease in the response amplitude of this biosensor compared to the control (without DMSO), which may indicate the influence of DMSO on the effectiveness of the hydrolysis reaction of the chromogenic marker ONPG by cytoplasmic bacterial β-galactosidase.

### 3.4. The Effect of Melittin on the lux-Biosensors

The cationic peptide Melittin is a toxic component of bee venom that can kill bacterial cells by disrupting their outer and inner membranes, as well as the peptidoglycan layer [40].

The effect of Melittin on the *lux*-biosensors *E. coli* MG1655 (pPpspA::lux) and *B. subtilis* 168 (pMW-PpspA) was studied in the concentration range of 0.2–20 µg/mL and 0.5–25 µg/mL, respectively. Melittin in the studied concentration ranges increases the bioluminescence intensity only of the *B. subtilis* 168 (pMW-PpspA) biosensor; the maximum luminescence induction factor (IF) was ~5 (Table 2).

**Figure 4:**
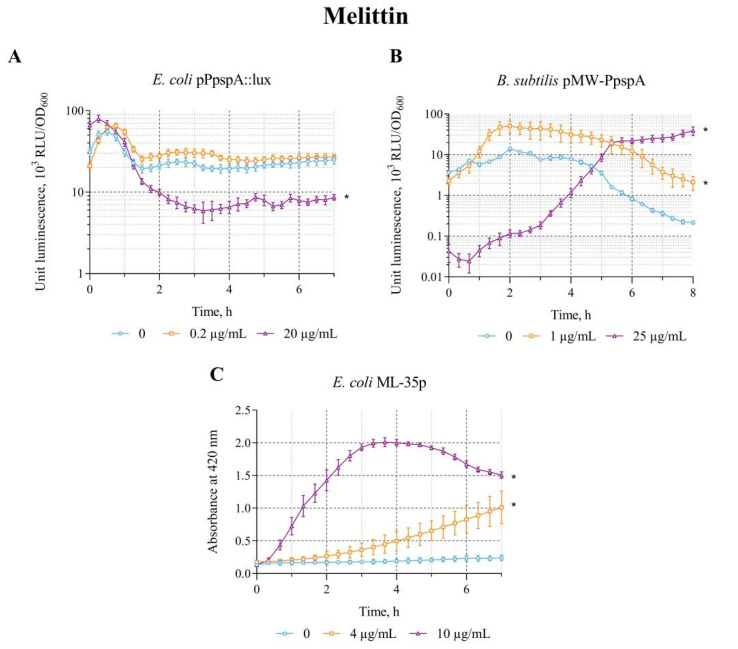
The response of the biosensor strains to Melittin. (**A**) *E. coli* MG1655 (pPpspA::lux), (**B**) *B. subtilis* 168 (pMW-PpspA), and (**C**) *E. coli* ML-35p. All values were mean ± standard deviations (SD) for n = 3 (biological replicates). Significant differences were determined using one-way ANOVA followed by Tukey’s HSD post hoc test (*p* ≤ 0.05). Asterisks (*) indicate significant differences compared to the control sample (without chem-icals).

A graphical representation of the response of the biosensor strains over time to different concentrations of Melittin is shown in Figure 4. Melittin (1 and 25 µg/mL) increases the bioluminescence intensity of *B. subtilis* 168 (pMW-PpspA). The response amplitude gradually increases up to an IF value of 4.5 and reaches a plateau after ~1.7 h (100 min) of incubation, with an induction start time of ~90 min at a Melittin concentration of 1 µg/mL. However, at a high concentration (25 µg/mL), it initially reduces the intensity of the bacterial luminescence (Figure 4B). Luminescence decreases by about an order of magnitude, and then begins to increase, crosses the control line (luminescence values of strain without Melittin) after ~4.5 h of incubation, and after 6 h of incubation exceeds the control line by ~21 times (Figure 4B). Melittin (0.2 μg/mL) in low concentration does not affect the luminescence intensity of *E. coli* MG1655 (pPpspA::lux)—the induction factor is less than2; a high concentration (20 μg/mL) even reduces the luminescence intensity of this biosensor by about 2–3 times after 1.3 h (80 min) of incubation (Figure 4A). Although different concentrations of Melittin do not induce the activity of the P*pspA* promoter in *E. coli* MG1655 (pPpspA::lux), its effect (in the concentration range of 1–10 μg/mL) enhances the permeability of the cytoplasmic membranes of *E. coli* ML-35p (Table 2). The response amplitude of the biosensor gradually increases up to an IF value of 10 at the induction start time of ~30 min at 10 μg/mL of Melittin (Figure 4C). Thus, the data obtained show that the effect of Melittin on biosensors is very similar to the effect of Triton X-100 (Figure 2 and Figure 4).

### 3.5. The Effect of Polymyxin B on the lux-Biosensors

Polymyxin B is a cyclic nonribosomal peptide produced by the bacterium *Paenibacillus polymyxa* that acts on bacterial membranes. It interacts with the lipopolysaccharide layer of the outer membrane of Gram-negative bacteria (with the phosphate groups of lipid A and the lipopolysaccharide core), which leads to the cell wall destruction [41,42].

The effect of polymyxin B on the biosensors *E. coli* MG1655 (pPpspA::lux), *B. subtilis* 168 (pMW-PpspA), and *E. coli* ML-35p was tested in the concentration ranges of 0.8–2 µg/mL, 0.5–5 µg/mL, and 1–10 µg/mL, respectively. The effect of polymyxin B is very similar to that of ethanol, which led to an increase in the response amplitude in all three biosensor strains. The IF in the studied concentration ranges is ~181, 6.4, and 3.3, respectively (Table 2).

**Figure 5:**
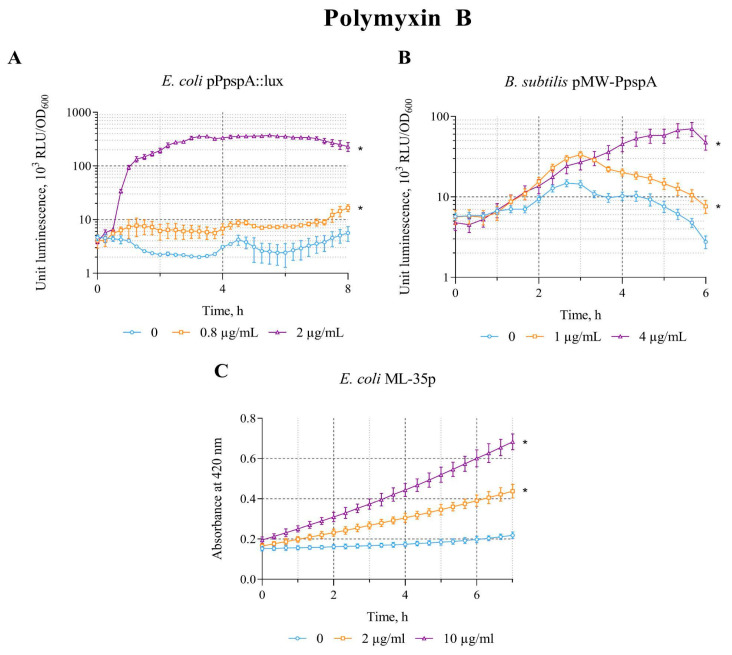
The response of the biosensor strains to polymyxin B. (**A**) *E. coli* MG1655 (pPpspA::lux), (**B**) *B. subtilis* 168 (pMW-PpspA), and (**C**) *E. coli* ML-35p. All values were mean ± standard deviations (SD) for n = 3 (biological replicates). Significant differences were determined using one-way ANOVA followed by Tukey’s HSD post hoc test (*p* ≤ 0.05). Asterisks (*) indicate significant differences compared to the control sample (without chem-icals). Summarized data on the influence of the studied compounds on the response of biosensor strains are presented in Table 3.

A graphical representation of the effect of polymyxin B on biosensors is shown in Figure 5. Polymyxin B (0.8 and 2 µg/mL) increased the intensity of bioluminescence of *E. coli* MG1655 (pPpspA::lux) strain. The response amplitude (IF) gradually increases up to an IF value of 160 and reaches a plateau after approximately3 h of incubation, with induction start time of ~45 min at a polymyxin B concentration of 2 µg/mL (Figure 5A). Exposure of *B. subtilis* 168 (pMW-PpspA) to polymyxin B at concentrations of 1 and 4 µg/mL also resulted in a gradual increase in bioluminescence. However, the response amplitude was weaker and the induction start time was longer (IST ~ 80 min) compared to the response of the *E. coli*-based *lux*-biosensor (Figure 5B). Furthermore, it was able to induce the permeability of *E. coli* ML-35p cytoplasmic membranes in the concentration range of 1–10 μg/mL (Table 2) with maximum response amplitude equal to 3. The response amplitude of the biosensor gradually increases by 2 and 3 times at 2 and 10 μg/mL of polymyxin B, respectively, and the induction start time is ~30 min (Figure 5C).

Thus, in this study, we demonstrated the functionality and efficiency of the constructed *lux*-biosensors (responding to membrane damage) and, using them, showed that some of the tested membrane-damaging agents are able to specifically activate stress response systems in the case of damage to membranes and cell walls.

## 4. Discussion

In this study, we constructed and tested whole-cell *lux*-biosensors for detecting bacterial response to membrane perturbations. Using the stress-inducible promoter of the *pspA* gene from *E. coli* and *B. subtilis*, respectively, fused with the *lux* gene cassette from *P. luminescens*, we demonstrated that these biosensors are capable of enhancing their luminescence in response to exposure to a number of known membrane-damaging compounds.

The *psp*A promoter was chosen to create the biosensor because *pspA* gene is known to be induced under various stress conditions, such as heat shock, infection by filamentous phages (production of secretin encoded by the phage, a protein forming pores in the outer membrane), inhibition of lipid biosynthesis and addition of proton ionophores, as well as the PspA protein (phage shock protein A) plays a key role in protecting the cell membrane from damage [31,43]. Moreover, Van Dyke previously suggested the possibility of using the *pspA* gene promoter to detect membrane damage in *E. coli* cells and demonstrated the induction of the *pspA-luxCDABE* reporter in the engineered *E. coli*-based biosensor during mechanical cell damage caused by acoustic cavitation resulting from exposure to high-frequency ultrasound waves [44].

The *B. subtilis* genome encodes two PspA paralogs: PspA and LiaH. The *pspA* gene is regulated by sigma factor σ^W^, which controls the transcription of genes involved in protecting the cell membrane from the penetration of antimicrobial peptides produced by Gram-positive bacteria. The *liaIHGFSR* operon encodes the integral membrane protein LiaI, which recruits LiaH following stress-mediated induction of this operon (the promoter P*lia*I is induced in response to a wide range of conditions that cause cell envelope stress, in particular, antibiotics that interfere with the membrane-anchored stages of cell wall biosynthesis). LiaIH proteins contribute to maintaining membrane integrity, as in the case of Psp response in Gram-negative bacteria. In addition, the use of whole-cell *lux*-biosensor based on P*liaI* of *B. subtilis* has been reported for the quantitative bioluminescent determination of cell wall integrity-damaging substances, including, but not limited to, antibiotics that disrupt the lipid II cycle [31,45].

The luminescent response of the constructed *lux*-biosensors was different under the action of the studied membrane-damaging agents. The luminescent response amplitude of the *E. coli* MG1655 (pPpspA::lux) strain was higher compared to the amplitude of the luminescent response of the *B. subtilis* 168 (pMW-PpspA) strain. The pPspA::lux plasmid has approximately a 4-fold higher copy number compared to the pMW-PpspA plasmid, which may contribute to the observed difference in the luminescent response of the strains. Although it is more likely that a higher copy number will enhance basal luminescence without rising IF. Another possible reason may be suboptimal cultivation conditions for *B. subtilis*, given the ability of the bacteria to form sporulation cells and the need to return the cells to a vegetative state. Therefore, several parameters of *B. subtilis* cultivation conditions were optimized (the dilution factor of the bacterial overnight culture and the incubation time of the diluted culture), which led to an approximately twofold increase in the response amplitude of the biosensor to tested compounds—the average value of the maximum induction factor became ~12. Even after this stage, the induction of the *pspA* promoter in *B. subtilis* cells is not as effective as in *E. coli* cells. The observed effect can be explained by the fact that *B. subtilis* has two *pspA* paralogs, *liaH* and *pspA*, which differ in genomic context and gene regulation, but exhibit functional similarities and may have at least partially overlapping functions related to protecting the bacterial envelope from damage [31,45]. Despite the fact that the luminescence response of the constructed *B. subtilis* 168 (pMW-PpspA) biosensor is lower than that of the *E. coli* MG1655 (pPpspA::lux) strain, its effectiveness is generally comparable to that of existing *lux*-biosensors based on *B. subtilis* designed to detect DNA-tropic agents and compounds causing oxidative stress [33].

Based on the data obtained, the following pattern of action of the studied compounds can be distinguished:

(1) Ethanol and polymyxin B are able to specifically activate stress response systems in case of damage to membranes and cell walls in both *E. coli* and *B. subtilis* cells as well as enhance the permeability/disruption of the inner membrane of *E. coli*.

Ethanol was used as a positive control, since it is known that it affects the *E. coli* membrane (e.g., it increases its permeability, causes changes in the lipid composition of the membrane, etc.) and strongly induces the *psp* operon in a dose-dependent manner [12,14,36,46,47]. The results showed that the effect of polymyxin B is very similar to that of ethanol. The data obtained are consistent with the model of the mechanism of bacterial destruction by polymyxin B (which targets lipopolysaccharides of both *E. coli* membranes), proposed by Borrelli K. et al., 2025 [48], and also probably indicate the involvement of *psp*-operon proteins, including PspA (which can directly interact with the inner membrane, stabilizing its structure and integrity), in protecting bacterial cells from membrane destruction caused by this antibiotic.

(2) Triton X-100 and melittin are able to specifically induce the stress-sensitive *pspA* promoter only in *B. subtilis* cells. Although these compounds do not specifically induce the P*pspA* promoter in *E. coli* MG1655 (pPpspA::lux), their action enhances the permeability/destruction of cytoplasmic membranes of *E. coli* ML-35p cells.

It is known that Melittin binds the bacterial membrane and folds into an α-helical structure at an angle that allows it to insert into the lipid bilayer [49]. This insertion creates pores on the membrane surface, increasing the permeability of the membrane barrier, allowing intracellular contents such as β-galactosidase to penetrate through it. Thus, when the cellular contents flow out (and/or ONPG penetrates the inner cell membrane and is cleaved by cytoplasmic β-galactosidase to o-nitrophenol), we observe the response of the *E. coli* ML-35p biosensor (a very fast exponential curve with a peak and subsequent decrease; Figure 4C). Although the outer membrane collapses rapidly, both membranes rapidly recompense shortly thereafter, leading to a temporary loss of integrity, followed by a stable state in which the membranes become impermeable again. In the studied concentration range, Melittin did not inhibit or only slightly inhibited bacterial growth. All this suggests that the damage caused by Melittin is not severe enough (there is no direct damage to membrane, disruption of the lipid bilayer and/or solubilization of membrane proteins) to activate the Psp system of *E. coli*. However, there is eventually a continuous weak leakage of cellular contents, which continues until the cell collapses, although this process can be slow and requires high concentrations of Melittin.

The action of Triton X-100 is somewhat similar to that of Melittin. It is also known that Triton X-100 penetrates into the lipid bilayer of cell membranes and, thus, like Melittin, creates holes, which leads to a violation of the compactness and integrity of the lipid membrane and allows water and macromolecules to penetrate into these holes, increasing the permeability of the cell membrane [37]. Thus, the effect of both Melittin and Triton X-100 in the studied concentration range is sufficient to lead (directly or indirectly) to an increase in the permeability of cell membranes, but the effect on the cell itself is apparently not strong enough to trigger the *E. coli* Psp stress response system.

The action of DMSO differs from other studied compounds and deserves to be mentioned. DMSO was able to induce the stress-sensitive *pspA* promoter only in *E. coli* cells, but not in *B. subtilis*. However, DMSO did not increase the permeability of the inner membrane of *E. coli*, but rather decreased it (Figure 3C). The observed effect was not related to the effect of DMSO on the hydrolysis reaction of the chromogenic marker ONPG by cytoplasmic bacterial β-galactosidase, since the action of DMSO (in the concentration range of 0.67–10%) on the supernatant of *E. coli* ML-35p cell-free lysate did not lead to a decrease in the enzymatic activity of β-galactosidase compared to the untreated control (Table 3). The data obtained are consistent with literature data showing that DMSO in high concentrations tends to slow down the enzymatic reaction rate, and therefore should always be used at a concentration below 10% for all permeabilization assays [50]. It can be assumed that the observed slower degradation of *E. coli* ML-35p cells in the presence of DMSO compared to the untreated control is partly related to the compound’s ability to induce the expression of the *psp* operon. This induction, in response to stress conditions caused by DMSO, in turn leads to the production of the PspA protein, which plays a key role in protecting and stabilizing the cell membrane due to its direct connection with the inner membrane [51]. It is known that DMSO is widely used as a solvent for water-insoluble substances, for cell-biological therapies and cryopreservation, particularly in cell culture laboratories. The effect of DMSO as a cryoprotector is due to its ability to quickly penetrate cell membranes, which promotes the displacement of water and postpone cell volume changes, reducing stress on the cells and protecting them from mechanical damage caused by the formation of ice crystals [39,52]. The data obtained suggest that the protective properties of DMSO for cells, in addition to those listed above, may also be associated with its ability to activate specific bacterial stress response systems (upon damage to membranes and cells), stabilizing the integrity of the inner membrane. Thus, it can be assumed that this biosensor set can be used, for example, to search for alternative cryoprotective agents with lower toxicity and impact on cellular functions. However, further research is needed to select the optimal biosensor set to identify patterns of action of these substances on bacterial cells.

Differences in induction of *pspA* promoters in *B. subtilis* and *E. coli* upon exposure to the same chemicals can be due to several factors. Firstly, the regulation of the *pspA* genes in these bacteria differs. The stress sensor in *E. coli* cells is the PspB-PspC complex, which, upon loss of PMF, undergoes conformational changes, binds to PspA, thus releasing PspF and allowing it to induce the *pspA* promoter. In *B. subtilis* cells, the sensor is the transmembrane protein RsiW, which, upon the onset of envelope stress, undergoes a series of proteolytic transformations and releases sigma factor σ^W^. Secondly, the structure and properties of cell membranes differ significantly. DMSO is a weak protonophore, causes a loss of PMF without inducing large-scale membrane damage, which leads to selective activation of *pspA* in *E. coli* but not in *B. subtilis*. Triton X100 and melittin can cause structural changes in the *B. subtilis* membrane that are pronounced enough to be detected by the RsiW system, but cause too short-term pore formation in the *E. coli* membranes to be detected by the PspB-PspC system. Thus, biosensors based on *E. coli* and *B. subtilis* complement each other, allowing to some extent to differentiate membrane damage by mechanism.

Thus, our study demonstrated the functionality and efficiency of the constructed *lux*-biosensors and, using them, it was shown that some of the tested compounds are able to specifically activate Psp stress response systems in case of membrane damage. The *lux*-biosensors we have designed can be used as part of a biosensor panel, expanding the range of tools for detecting specific stress responses (such as oxidative stress, heat shock, and DNA and membrane damage) in bacterial cells.

In addition to the *lux*-biosensors we have designed, there are other sensors for detecting membrane stress responses. In *E. coli*, the genes activated in response to membrane perturbations, in addition to the *psp* genes, include genes involved in the biosynthesis of fatty acid (components of cell membranes). In this regard, *lux*-biosensors have been developed that use the promoters of these genes to detect toxic substances that damage lipids and membranes. For example, a biosensor based on the *fabA* gene promoter, the luminescence of which was induced by treatment of cells with compounds that disrupt the membrane structure (e.g., phenol, detergents Triton X100, Tween-80, sodium dodecyl sulfate), or antibiotics that inhibit fatty acid biosynthesis (e.g., cerulenin). However, the authors emphasized that the *fabA lux*-biosensor is more suitable for determining general toxicity, since it can detect the toxicity of various hazardous chemicals, including DNA- and oxidative-damaging agents [9,44]. Shideler and co-authors used the *pmr*/*spe* promoter to create *lux*-biosensors based on *Pseudomonas aeruginosa* to detect compounds causing damage to the outer membrane. These biosensors can serve as a convenient tool for the discovery of new antimicrobial drugs that specifically target the outer membrane. Some of the disadvantages of such biosensors include the fact that the promoters used (*pmr*, *spe*, as well as our *pspA*) can be induced under various stressful environmental conditions, which raise questions about their specificity and requires further additional research [53].

As emphasized in the works of Belkin et al. [54], the use of living sensory cells as analytical tools has a number of advantages. The use of whole-cell biosensors can provide answers to questions such as “how toxic is the sample” or “how mutagenic is the chemical.” A living cell can provide data on the bioavailability of a pollutant compared to the total concentration of that chemical in a sample. Another inherent advantage of using live bioreporters is the technical possibility of using them in schemes in which the sensing agent (i.e., the live biosensor) is spatially separated from the signal recording equipment. An example of this type of application is the remote detection of buried explosives, when bioluminescent bacteria sensitive to DNT/TNT are scattered on the ground, and imaging equipment is located remotely. Bioluminescent reporter strains have also been used to study the antibacterial effects and mechanism of action of CdSe quantum dots, rice seedling exudates or UV radiation. Such sensors also played a crucial role in detecting the DNA-damaging effect of lyophilization (a common method of long-term preservation of microbial cultures) and in studying the bactericidal effect of disinfectants (e.g., hypochlorous acid) [54]. However, the practical use of *lux*-biosensors may face challenges, as issues often arise related to the release of genetically modified microorganisms into the environment. As Chemla and coauthors recently noted [55], these problems have serious regulatory and societal implications that need to be addressed.

## Figures and Tables

**Table 1:** Plasmids and bacterial strains used in the study.

Name	Description	Source/Reference
Bacterial strains		
*E. coli* K12 MC1061	F-D(araA-leu)7697 [araD139]B/r A(codB-lacI)3 galK16 galE15(GalS) A-e14-mcrA0 relA1 rpsL150 spoT1 mcrB1 hsdR2	VKPM (Moscow, Russia)
*E. coli* K12 MG1655	F-ilvG rfb-50 rph-1	VKPM (Moscow, Russia)
*E. coli* K12 ML-35p	*lacI^−^ lacY^−^ lacZ^+^*	[21]
*B. subtilis* 168	trpC2	VKPM (Moscow, Russia)
Plasmids		
pPL_ABCDExen-cat-hairpin	A promoterless shuttle vector with *luxABCDE* genes from *P. luminescens*. Two replication origins (from pMW118 and pBS72). A low copy number plasmid (6 units per chromosome). Resistance to trimethoprim (Tp^r^), chloramphenicol (Cm^r^) and ampicillin (Ap^r^)	This study
pMW-PpspA	The pPL_ABCDExen-cat-hairpin vector with the insertion of the *B. subtilis* P*pspA* promoter; P*pspA* is transcriptionally fused with *luxABCDE* genes from *P. luminescens*	This study
pDEW201	Promoter-probe vector with *luxCDABE* genes from *P. luminescens*. A moderate copy number plasmid. Ap^r^	[22]
pPpspA::lux	The *E. coli* P*pspA* promoter was cloned into the pDEW201 vector and transcriptionally fused with *luxCDABE* reporter genes from *P. luminescens*. Ap^r^	This study

**Table 2:** Comparison of the *E. coli* MG1655 pPspA::lux and *B. subtilis* 168 pMW-PpspA biosensors’ response to stress caused by membrane damage.

Toxicant	Biosensor Characteristic	*E. coli* MG1655 (pPpspA::lux)	*B. subtilis* 168 (pMW-PpspA)	*E. coli* ML-35p
	Threshold concentration, %	0.4	0.5	2.0
Ethanol	Induction factor (IF) *	34.0 ± 3.0	4.8 ± 1.5	3.3 ± 1.0
	Concentration range, %	0.4–8.0	0.5–5.0	1.0–10.0
	Induction start time, min	150	30	30
	Threshold concentration, µg/mL	n/a	20	n/d
Triton X-100	Induction factor	n/a	6.9 ± 1.4	5.9 ± 0.5
	Concentration range, µg/mL	40–400	20–200	133–4000
	Induction start time, min	n/a	30	45
	Threshold concentration, %	1.0	n/a	n/a
DMSO	Induction factor	9.6 ± 3.6	n/a	n/a
	Concentration range, %	0.5–5.0	0.25–2.5	0.67–10
	Induction start time, min	110	n/a	n/a
	Threshold concentration, µg/mL	n/a	1.0	2.0
Melittin	Induction factor	n/a	4.8 ± 1.7	10.0 ± 0.8
	Concentration range, µg/mL	0.2–20.0	0.5–25.0	1.0–10.0
	Induction start time, min	n/a	90	30
	Threshold concentration, µg/mL	0.8	1.0	2.0
Polymyxin B	Induction factor	180.7 ± 9.2	6.4 ± 2.4	3.3 ± 0.7
	Concentration range, µg/mL	0.8–2.0	0.5–5.0	1.0–10.0
	Induction start time, min	45	80	30

n/a—“not applicable”, the compound does not induce luminescence of biosensor cells in any concentrations; n/d—“not determined”, the threshold concentration has not been determined; * the average value of maximum IF is presented.

**Table 3:** The effect of the studied compounds on the biosensor response.

Biosensor Strains	Tested Compound				
	Ethanol	Polymyxin B	Triton X-100	Melittin	DMSO
*E. coli* MG1655 (pPpspA::lux)	*++*	*++*	*-*	*-*	*+*
*B. subtilis* 168 (pMW-PpspA)	+	+	+	+	-
*E. coli* ML-35p	+	+	+	++	-
*E. coli* ML-35p cell-free lysate *	n/a	n/a	n/a	n/a	n/a

“++”—the biosensor response amplitude increases more than 10 times when exposed to the test compound (compared to the untreated control); “+”—the biosensor response amplitude increases more than 2 times when exposed to the test compound (compared to the untreated control); “-”—the value of biosensor response amplitude less than 2 when exposed to the test compound (compared to the untreated control); * supernatant of *E. coli* ML-35p cell-free lysate, used to evaluate the effect of the studied compounds (ethanol—1–10%, Polymyxin B and Mellitin—1–10 μg/mL, DMSO—0.67–10%, and Triton X-100–400 μg/mL) on the hydrolysis reaction of the chromogenic marker ONPG by cytoplasmic bacterial β-galactosidase; n/a—the test compound does not affect the enzymatic activity of β-galactosidase (compared to the control sample without the studied compound).

## Data Availability

Data is contained within the article or Supplementary Materials.

## Supplementary Materials

The following supporting information can be downloaded at: https://www.mdpi.com/article/10.3390/bios15120780/s1, Figure S1: The response of the *lux*-biosensor strains to the studied chemicals; Figure S2: The effect of the studied chemicals on the luminescence intensity of strains (**A**) *E. coli* MG1655 (pDlac) and (**B**) *B. subtilis* 168 (pPfbaA_ABCDExen); Figure S3: The response of the *lux*-biosensor strains to the hydrogen peroxide, paraquat, and anhydrotetracycline.

## Abbreviations

The following abbreviations are used in this manuscript:

RBS	ribosome binding site
DMSO	Dimethyl sulfoxide
ONPG	o-nitrophenyl-3-D-galactoside
IF	induction factor
PQ	paraquat
PMF	proton motive force

## Appendix A. Construction of Plasmids

Primers used in this work and intermediate plasmids used to obtain plasmid pMW-PpspA are listed in Table A1.

The pMWAL1T-Ppur plasmid contains the pBS72 and pMW118 replicons, as well as chloramphenicol and ampicillin resistance markers active in *B. subtilis* and *E. coli*, respectively [27]. This plasmid was used as a template for long-range PCR using the P1/P2 primer pair to remove the chloramphenicol resistance marker, resulting in a 6374 bp DNA fragment obtained by long-range PCR. The resulting DNA fragment was treated using the NcoI restriction enzyme and ligated into a circle. The ligase mixture was transformed into *E. coli* strain MC1061 with selection on ampicillin. The selection of clones was carried out by PCR screening for the absence of a fragment encoding the chloramphenicol resistance gene. Plasmid DNA, designated pMWAL_Ppur_cat-del, was isolated from the selected clone and confirmed by analytical restriction with the endonuclease NcoI in comparison with the original plasmid pMWAL1T-Ppur.

A DNA fragment containing the *lux* gene cassette, the trimethoprim resistance marker, and the T1T2 terminators of the *rrn*B gene, 8271 bp in size, was excised from the promoterless plasmid pPL_ABCDExen [27] at the SacI/SpeI restriction sites and ligated with a 4812 bp SacI/SpeI fragment from the plasmid pMWAL1T_Ppur_cat-del, resulting in the plasmid pMWAL1T_Ppur_cat-del_luxABCDE, 13083 bp in size. The resulting plasmid was tested by analytical restriction.

The pMWAL1T-Ppur plasmid was used as a template for PCR using the P3/P4 primer pair to amplify a 753 bp DNA fragment containing the structural part of the chloramphenicol resistance gene. This fragment was cloned by Gibson assembly into the plasmid pMWAL1T_Ppur_cat-del_luxABCDE, pre-treated with the restriction endonuclease BamHI and ApaI. As a result of cloning, the plasmid pPL_ABCDExen-cat was obtained, containing the chloramphenicol resistance gene located after the *lux* gene cassette, as well as with added SmaI restriction site and removed BamHI site.

A 112 bp DNA fragment obtained by PCR amplification of primer dimers using primers P5/P6 was inserted into the pPL_ABCDExen-cat plasmid at the XhoI restriction site using Gibson assembly.

This fragment contains Kpn2I and BamHI restriction sites, six stop codons (two in each of the three frames towards the *lux* gene cassette), and a hairpin structure upstream of the RBS, which is necessary to shield the potential influence of cloned promoter-containing fragments of 5′-terminal nucleotides on the formation of the RBS structure of the reporter gene (*lux*A) at subsequent stages.

The constructed plasmid was named pPL_ABCDExen-cat-hairpin (a low copy number plasmid, 6 units per chromosome [56]) and was used to obtain the final pMW-PpspA plasmid (Table 1). Transformation of *Bacillus subtilis* 168 with the pMW-PpspA plasmid was carried out using the Spizizen method [25].

Figure A1 shows the structure of the pMW-PpspA plasmid.

**Table A1:** Primers and intermediate plasmids used in this work.

Primer	Nucleotide Sequence		
P1	GCTCCATGGCTTTTATAATATGAGATAATGCCGACTGTACTTT		
P2	GCTCCATGGCCCACTTTATCCAATTTTCGTTTGTTGAACTA		
P3	TTTCACTTCTGAGTTCGGCATGGGCGGCGCGCCGGGCCCGTCGGCATTATCTCATATTATAAAAGCCAGTC		
P4	CCGAAGCGTTTGATAGTTAAGTCGACCCGGGTAGGAGGCATATCAAATGAACTTTAATAAAATTGATTTAG		
P5	TCCTCTTGCTTAGTTATCCGGATTAGGTTAGCTTACAACTGGGTGCAATTCTGC		
P6	CAGGAATTCGAGCTCGGTACCGCGGCCGCTCGAGCCGGATCCCCAACGTGAACTGGGTGCAGAATTGCACCCAGTTGTAAG		
P7	GTTCTCGAGTTTAAGCCTTGTCCAATTAAGCATTGATATTC		
P8	ATTGGTACCTGGAGCGATTGATGCACTGCCG		
pspAF	GCTGGATCCGATGAAATTCGCCACTTGTT		
pspAR	GGTGGTACCAATGTTGTCCTCTTGATTTC		
**Plasmids ***			
**Name**		**Description**	**Source/Reference**
pPL_ABCDExen		Intermediate plasmid (*lux* gene cassette, the trimethoprim resistance marker, and the T1T2 terminators donor) in the construction of the pPL_ABCDExen-cat-hairpin vector. A promoterless shuttle vector with *luxABCDE* genes from *P. luminescens*. The order of genes in the *lux* gene cassette and RBS upstream of each gene are optimized for *B. subtilis* expression. Two replication origins (from pMW118 and pBS72). Resistance to trimethoprim (Tp^r^), chloramphenicol (Cm^r^) and ampicillin (Ap^r^).	[27]
pMWAL1T-Ppur		Helper plasmid for construction of pPL_ABCDExen-cat-hairpin. Two replication origins (from pMW118 and pBS72). Resistance to chloramphenicol (Cm^r^) and ampicillin (Ap^r^).	[27]
pMWAL_Ppur_cat-del		Intermediate plasmid in the construction of the pPL_ABCDExen-cat-hairpin vector. Two replication origins (from pMW118 and pBS72). Resistance to ampicillin (Ap^r^).	This study
pMWAL1T_Ppur_cat-del_luxABCDE		Intermediate plasmid in the construction of the pPL_ABCDExen-cat-hairpin vector. A promoterless shuttle vector with *luxABCDE* genes from *P. luminescens*. Two replication origins (from pMW118 and pBS72). Resistance to trimethoprim (Tp^r^) and ampicillin (Ap^r^).	This study
pPL_ABCDExen-cat		Intermediate plasmid in the construction of the pPL_ABCDExen-cat-hairpin vector. A promoterless shuttle vector with *luxABCDE* genes from *P. luminescens*. Two replication origins (from pMW118 and pBS72). Resistance to trimethoprim (Tp^r^), chloramphenicol (Cm^r^) and ampicillin (Ap^r^).	This study
pPL_ABCDExen-cat-hairpin		A promoterless shuttle vector with *luxABCDE* genes from *P. luminescens*. Two replication origins (from pMW118 and pBS72). Resistance to trimethoprim (Tp^r^), chloramphenicol (Cm^r^) and ampicillin (Ap^r^).	This study

*****—intermediate plasmids used to obtain the final plasmid pMW-PpspA introduced into the *B. subtilis.*
