# Supplementary material for: Bacterial lux-Biosensors for Detecting Specific Cell Responses to Membrane Damage
Source: Biosensors (Basel). 2025 Nov 26;15(12):780. doi: 10.3390/bios15120780 (PMC12731034; doi:10.3390/bios15120780)
Supplement: Supplementary file 1 [file biosensors-15-00780-s001.zip › biosensors-3944315-supplementary.pdf]

Bacterial *lux*-biosensors for detecting specific cell responses to membrane damage

Supplementary Materials

The luminescence of *lux*-biosensors in the presence of various concentrations of ethanol, Triton X100, DMSO, polymyxin B, and melittin is shown in Figure S1. The graphs show the dose-dependent responses of the *E. coli* MG1655 (pPpspA::lux) and *B. subtilis* 168 (pMW-PpspA) biosensors to the studied chemicals.

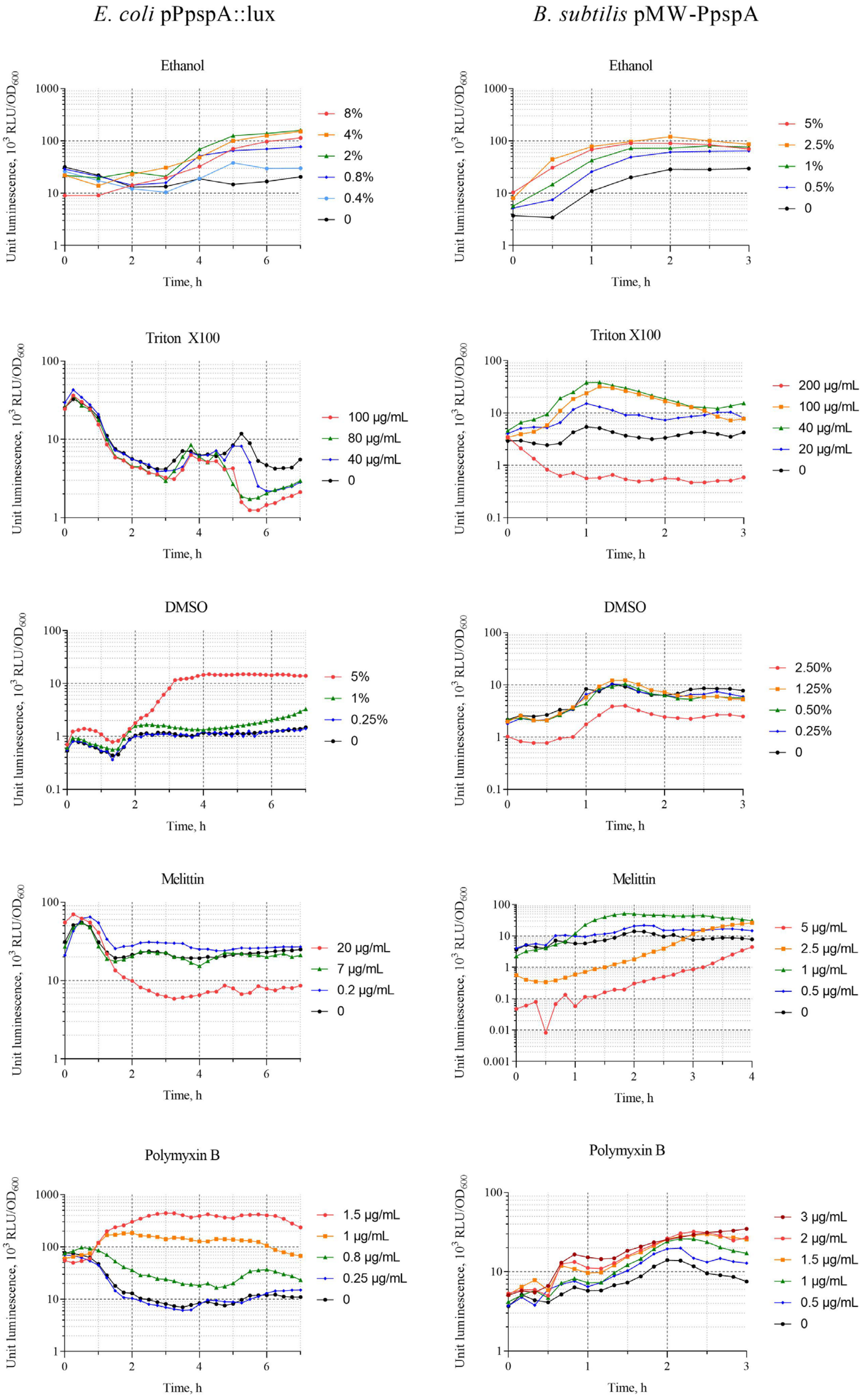

Figure S1. The response of the *lux*-biosensor strains to the studied chemicals.

(**A**) *E. coli* MG1655 (pPpspA::lux) and (**B**) *B. subtilis* 168 (pMW-PpspA). The curves show the change in luminescence units of the biosensor cell response over time, which is expressed as the ratio of the average values of light emission (in Relative Light Units, RLU) measured at time *t* to its optical density (OD_{600}) in the presence of various concentrations of ethanol, Triton X-100, DMSO, Melittin, and polymyxin B.

The ability of the studied chemicals to influence an *in vivo* bacterial bioluminescence was assessed using strains *E. coli* MG1655 and *B. subtilis* 168 carrying plasmids pDlac and pPfbaA_ABCDExen, correspondingly, with *lux* gene cassettes under the control of constitutive promoters. The plasmid pDlac is the pDEW201 derivative with transcriptional fusion of synthetic operator-less *E. coli* P*lac* promoter and *luxCDABE* reporter genes from *P. luminescens* [32]. The plasmid pPfbaA_ABCDExen contains bioluminescent *luxABCDE* reporter genes under the control of the constitutive P*fbaA* promoter in *B. subtilis* 168 [27]. Ethanol, DMSO, Triton X100, Polymyxin B, and Melittin were added to these strains at a concentration taken from the range of concentrations studied on *lux*-biosensors with a stress-inducible promoter of the *pspA* gene.

The addition of the studied chemicals did not lead to an increase in the luminescence of *E. coli* MG1655 (pDlac) and *B. subtilis* 168 (pPfbaA_ABCDExen) strains (Fig. S2).

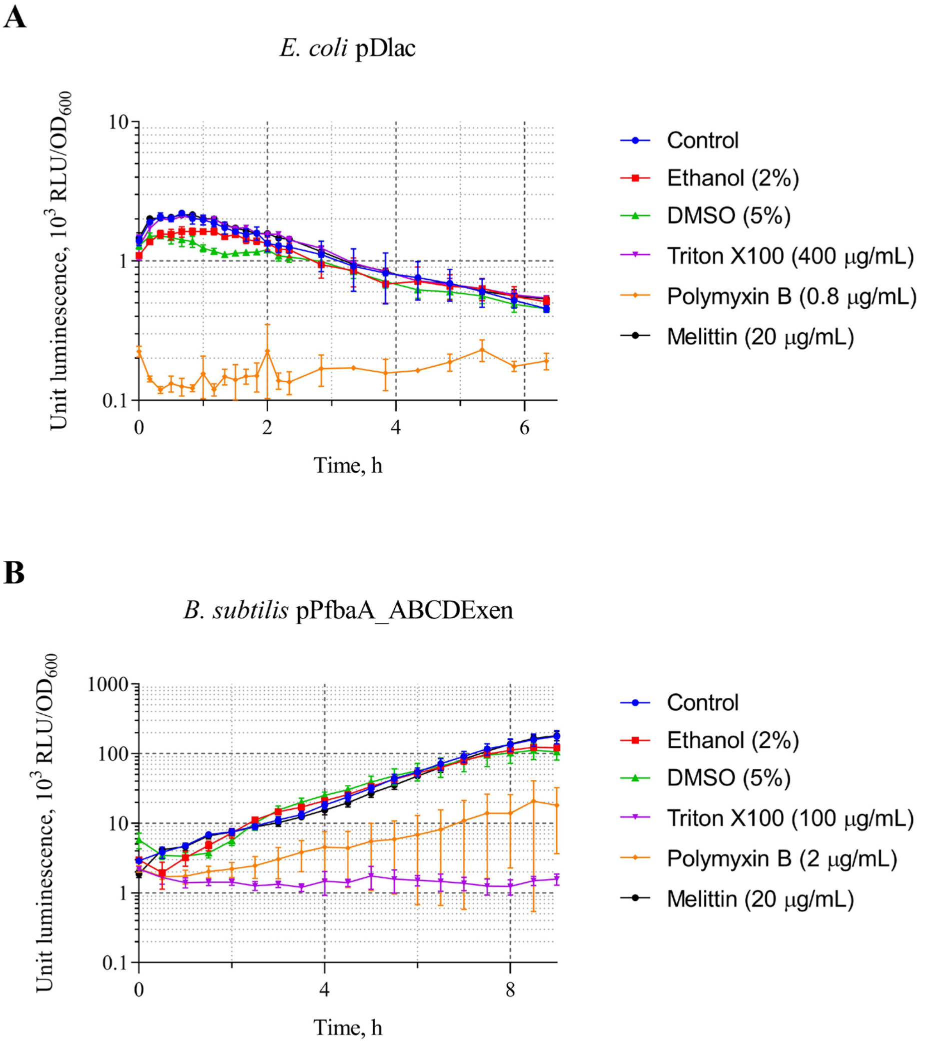

Figure S2. The effect of the studied chemicals on the luminescence intensity of strains (**A**) *E. coli* MG1655 (pDlac) and (**B**) *B. subtilis* 168 (pPfbaA_ABCDExen).

(**A,B**) - The curves show the change in luminescence units of the bacterial cell response over time, which is expressed as the ratio of the average values of light emission (in RLU) measured at time *t* to its optical density (OD_{600}) in the presence of studied chemicals. The control measurements ("Control") were carried out without the use of chemicals. All values were mean ± standard deviations (SD).

In addition, the specificity of the constructed *lux*-biosensors (*E. coli* MG1655 (pPpspA::lux) and *B. subtilis* 168 (pMW-PpspA)) was tested using agents that cause oxidative damage, such as hydrogen

peroxide and paraquat, as well as the antibiotic anhydrotetracycline (binds to the bacterial ribosome, inhibiting protein synthesis and preventing the translation process) as a negative controls. These chemicals were added to these strains in a concentration that maximally enhanced the luminescence of specific *lux*-biosensors for detecting oxidative stress [26,45] and tetracycline antibiotics [33].

The addition of these chemicals did not activate the luminescence of the *E. coli* MG1655 (pPpspA::lux) and *B. subtilis* 168 (pMW-PpspA) *lux*-biosensors (Fig. S3), which means that their action did not lead to specific induction of the stress-sensitive *psp*A gene promoter.

A

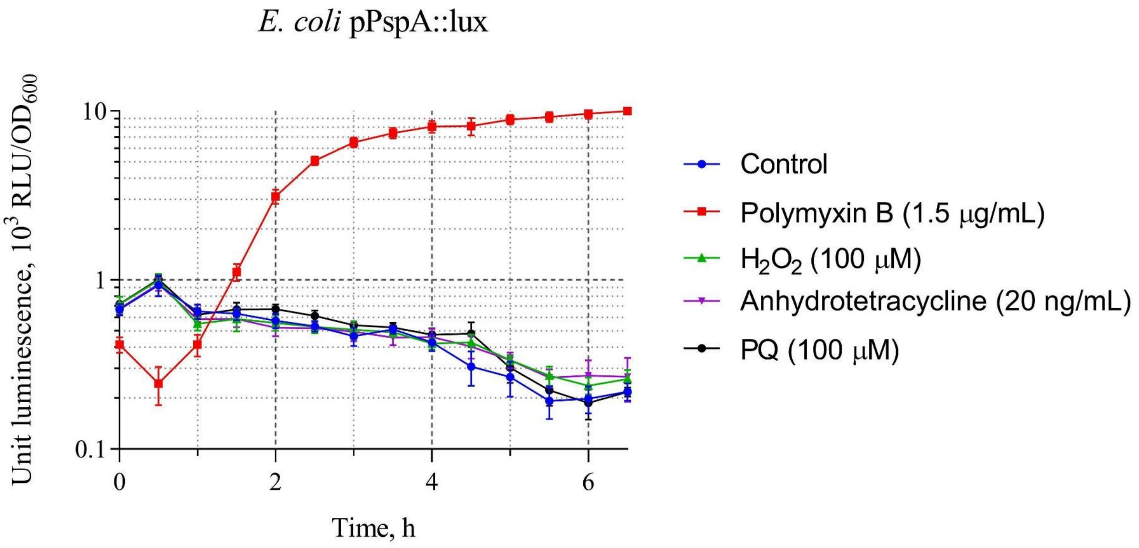

B

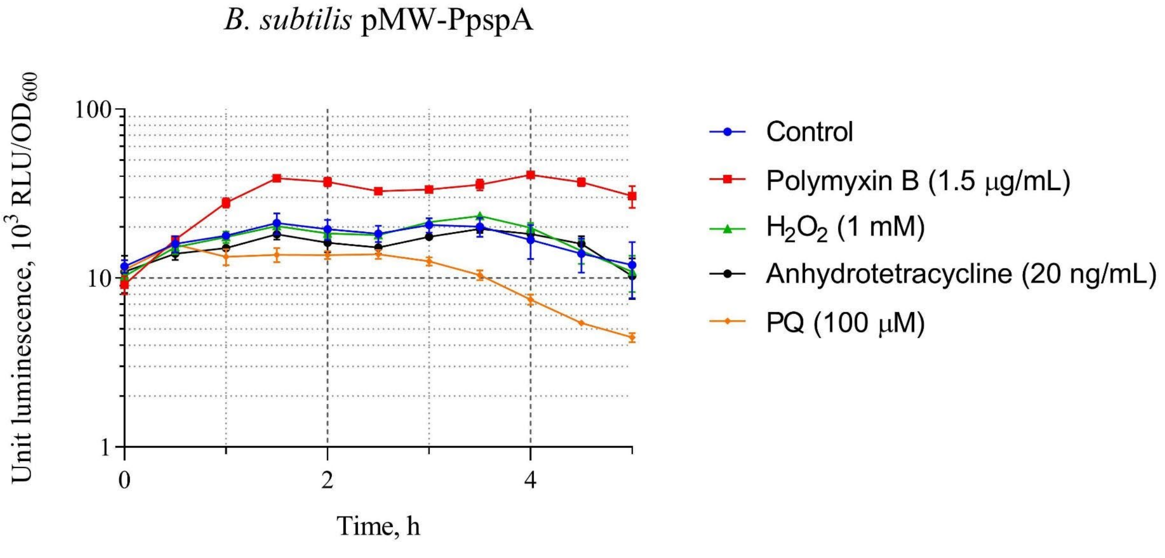

Figure S3. The response of the *lux*-biosensor strains to the hydrogen peroxide, paraquat, and anhydrotetracycline. (**A**) *E. coli* MG1655 (pPpspA::lux) and (**B**) *B. subtilis* 168 (pMW-PpspA). (**A,B**) - The curves show the change in luminescence units of the bacterial cell response over time, which is expressed as the ratio of the average values of light emission (in RLU) measured at time *t* to its optical density (OD_{600}) in the presence of the studied chemicals. The control measurements ("Control") were carried out without the use of chemicals. All values were mean ± standard deviations (SD). Polymyxin B was used as a positive control.
